# Spatiotemporal trends in tuberculosis incidence in Thailand, 2012–2023: a nationwide, province-level analysis

**DOI:** 10.1186/s40249-026-01473-2

**Published:** 2026-07-01

**Authors:** Kittipong Sornlorm, Roshan Kumar Mahato, Sarayu Muntaphan, Kanit Hnuploy, Rajitra Nawawonganun

**Affiliations:** 1 Faculty of Public Health, Khon Kaen University, Khon Kaen, Thailand; 2 Khon Kaen Provincial Health Office, Khon Kaen, Thailand; 3 Faculty of Science and Technology, Suratthani Rajabhat University, Surat Thani, Thailand

**Keywords:** Tuberculosis, Thailand, Spatiotemporal analysis, Disease mapping, Epidemiology, Health policy, Joinpoint regression

## Abstract

**Background:**

Tuberculosis (TB) remains a major public health challenge in Thailand, a high-burden country undergoing both epidemiological transition and pandemic-related disruption. This study examined temporal and spatial patterns of age-standardized TB incidence from 2012 to 2023 across Thailand’s 13 health regions and 77 provinces.

**Methods:**

This nationwide ecological study used annual TB case notifications (2012–2023) for all 77 Thai provinces from Thailand's National Disease Surveillance System (Report 506), Bureau of Epidemiology, with mid-year and age-stratified provincial populations. Age-standardized incidence rates (ASR) were calculated using the WHO World Standard Population (2000–2025). Temporal trends were assessed by Joinpoint regression with Bayesian-Information-Criterion model selection (maximum 2 joinpoints) and validated by generalized additive models. Spatial clustering was evaluated by Global Moran’s *I* and Local Indicators of Spatial Association (LISA) under queen contiguity, with sensitivity analyses across alternative weights and Benjamini–Hochberg false-discovery-rate correction. Provinces were classified into long-term trend categories combining effect size (AAPC) and significance, and COVID-19 impact was quantified by 2019–2023 ASR percent change.

**Results:**

National TB incidence declined substantially from 2012 to 2023, although marked regional heterogeneity persisted. Five health regions showed the strongest long-term reductions: region 3 [AAPC: − 20.51, 95% confidence interval (*CI*)*:* − 33.00 to − 13.77], region 9 (− 19.42, 95% *CI:* − 28.98 to − 8.57), region 4 (− 17.53, 95% *CI:* − 23.64 to − 12.89), region 11 (− 11.79, 95% *CI:* − 22.16 to − 0.04), and region 13 (− 11.19, 95% *CI:* − 22.44 to − 3.74). Region 6 showed an early decline followed by stabilisation, and region 12 showed a mid-period increase before a post-2019 reduction.

**Conclusions:**

Thailand has achieved substantial overall reductions in TB incidence over the past decade, but pronounced regional and provincial disparities persist. Localized hot-spots, biphasic regional trajectories that may reflect surveillance and programmatic transitions, and compound vulnerability during the COVID-19 period underscore the need for geographically targeted monitoring, equitable resource allocation, and pro-poor interventions to sustain progress toward TB elimination.

**Supplementary Information:**

The online version contains supplementary material available at 10.1186/s40249-026-01473-2.

## Background

Tuberculosis (TB) remains one of the leading causes of morbidity and mortality worldwide, representing a persistent global health crisis. In 2022 alone, an estimated 10.6 million new cases and 1.3 million deaths were attributed to the disease [1–3]. Despite significant progress in healthcare infrastructure and disease control in recent decades, Thailand remains classified among the 30 high-burden countries for TB by the World Health Organization (WHO) [3]. To address this, the WHO End TB Strategy launched in 2015 established ambitious targets including a 90% reduction in TB incidence and a 95% reduction in TB deaths by 2035 relative to 2015 levels [4]. However, achieving these targets requires navigating complex epidemiological landscapes and unexpected global disruptions.

The emergence of the COVID-19 pandemic profoundly disrupted global TB control programs, resulting in estimated declines of 25–40% in TB notifications during lockdown periods [5, 6]. In Thailand, the pandemic placed immense strain on the healthcare system, necessitating a rapid reallocation of resources and creating disparities in healthcare accessibility. Previous research on the spatial distribution of COVID-19 health resources in Thailand highlighted significant inequalities in service coverage during the crisis, suggesting that the pandemic’s impact was geographically uneven [7]. Reduced access to diagnostic services, healthcare system strain, and socioeconomic impacts created conditions that may have facilitated TB transmission while simultaneously lowering detection capacity. Understanding these pandemic-related disruptions through a spatial lens is critical for recovery planning and future preparedness.

Spatial epidemiology provides powerful tools for understanding geographic heterogeneity in disease burden and identifying high-risk areas requiring intervention [8, 9]. Moving beyond national averages is essential, as TB transmission commonly clusters in specific "hotspots" driven by local factors. Recent studies involving spatiotemporal analysis in other high-burden settings have demonstrated the usefulness of these methods. For instance, Kulldorff’s spatiotemporal scan statistics and discrete Poisson models have successfully identified persistent TB clusters and their geographic distribution, allowing for more precise resource prioritization [10]. TB disproportionately affects vulnerable and marginalized populations, particularly those living in poverty. In Thailand, geographic disparities in TB burden mirror broader patterns of socioeconomic inequality, with higher incidence concentrated in border regions, migrant communities, and areas with limited healthcare access. Understanding these spatial patterns is essential for designing pro-poor, equity-focused interventions.

This study applied these advanced spatial methodologies to the Thai context. Spatial autocorrelation methods, including Global Moran’s *I* and Local Indicators of Spatial Association (LISA), are employed to detect clustering patterns that inform resource allocation and targeted prevention strategies [11, 12]. Complementing this, Joinpoint regression analysis is used to identify significant changes in epidemiological trends and to estimate annual percent change (APC) for distinct temporal segments [13, 14]. This approach enables the detection of inflection points such as those associated with the implementation of the End TB Strategy or the onset of COVID-19 and provides insights into program effectiveness and external influences on TB epidemiology.

This study aimed to: (1) characterize temporal trends in TB incidence across Thailand’s health regions and 77 provinces from 2012 to 2023 using Joinpoint regression; (2) assess spatial autocorrelation and clustering patterns using Global Moran’s *I* and LISA; (3) classify provinces into risk categories based on long-term trends using a five-level effect-size-and-significance scheme combining AAPC magnitude (± 5%) and statistical significance (*P* < 0.05), reported alongside the conventional binary Safe/Alarming Zone classification; (4) quantify the COVID-19 pandemic’s impact on provincial TB rates through both crude- and age-standardized rate percent change between the immediate pre-pandemic baseline (2019) and the post-pandemic recovery year (2023), categorised as Decreased (< − 5%), Unchanged (± 5%), or Increased (> + 5%); and (5) identify priority areas for targeted intervention. Findings will inform evidence-based, geographically targeted strategies to accelerate progress toward TB elimination goals.

## Methods

### Study design and setting

This study conducted a retrospective ecological study analyzing tuberculosis incidence across all 77 provinces of Thailand from 2012 to 2023. Thailand is administratively divided into 13 health regions comprising 77 provinces. This study utilized population-level surveillance data to characterize temporal trends, spatial patterns, and pandemic-related disruptions in TB epidemiology.

### Data sources

TB case data: Monthly TB case notifications (2012–2023) were obtained from the National Disease Surveillance System (Report 506), Bureau of Epidemiology, Department of Disease Control, Ministry of Public Health, Thailand [15]. This system captures all TB cases diagnosed and reported throughout public and private healthcare facilities. Inclusion criteria for this analysis comprised all incident TB notifications (both bacteriologically confirmed and clinically diagnosed cases of all anatomical sites and all ages) reported to the Report 506 surveillance system between 1 January 2012 and 31 December 2023. Recurrent episodes within an individual were treated as separate notification events, consistent with the WHO recording-and-reporting framework and Thailand’s National TB Programme practice. Drug-resistant TB cases were not excluded because the Report 506 platform aggregates resistance status under the same case definition; however, sensitivity analyses excluding suspected drug-resistant notifications produced concordant temporal patterns. Cases with missing date of diagnosis, unknown province of residence or implausible age (> 110 years) were excluded (< 0.3% of the original records).

Population data: Annual mid-year population estimates by province, and age group were obtained from the Official Statistics Registration Systems, Department of Provincial Administration, Ministry of Interior Thailand [16]. Age-specific population data were aggregated into 5-year age groups (0–4, 5–9, …, 85 +) for age standardization.

WHO Standard Population: The WHO World Standard Population (2000–2025) [17] was used as the reference for age standardization to enable international comparability.

Geographic boundaries: Provincial administrative boundary shapefiles were obtained from the UN Office for the Coordination of Humanitarian Affairs (OCHA) Thailand database [18] for spatial analysis.

### Age-standardized rate (ASR) and standard error (SE) calculation

Age-standardized incidence rates (ASR) were calculated using the direct standardization method with the WHO World Standard Population (2000–2025) as reference. The ASR formula is:

$$ASR=(\Sigma ({a}_{i}\times {w}_{i}) /\Sigma {w}_{i})\times \mathrm{100,000}$$

where *a*ᵢ is the age-specific incidence rate in age group i, and *w*ᵢ is the proportion of the standard population in age group i. Age groups were categorized into 19 groups (0–4, 5–9, …, 85–89, 90 +). The standard error (SE) of ASR was calculated using [19]:

$$SE(ASR)=\sqrt{\Sigma ({w}^{{i}^{2}}\times {a}_{i}/{n}_{i})}/\Sigma {w}_{i}\times \mathrm{100,000}$$

where *n*ᵢ is the population in age group i. For province-year observations with zero TB cases in any of the 19 age strata, we applied the gamma-method exact correction described by Fay and Feuer [19], implemented by adding 0.5 to the empirical numerator (*a*₀.₅ = (Cases + 0.5)/Population × 100,000) before age weighting. Across the 924 province-year records, 158 observations (17.1%) contained at least one age stratum with zero notifications; these clustered predominantly in low-population provinces (median population 518,574; range 170,226–5,491,302) and during the early surveillance period (2012–2016 accounted for 65.8% of zero records, while 2020–2022 contributed only 14.6%). The mean rate inflation introduced by the + 0.5 correction was 0.12 per 100,000 (range 0.01–0.30), negligible relative to the observed ASR distribution. Sensitivity analyses replacing zero-case records with three alternative imputations (a) leaving zero values unchanged, (b) + 1.0 continuity correction, and (c) Bayesian gamma–Poisson posterior mean with weakly informative prior produced AAPC estimates for all 13 health regions that were within ± 0.6 percentage points of the primary analysis (Supplementary Table S2), confirming that the + 0.5 correction did not materially bias the temporal trend estimates.

### Joinpoint regression analysis and generalized additive models: APC and AAPC

Temporal trend analysis was performed using Joinpoint regression (Joinpoint Regression Program, Version 5.4.0.0) [14]. This method fits a series of joined straight lines on a log scale to identify significant changes in temporal trends. The annual percent change (APC) for each linear segment is calculated as:

$$APC=[exp(\beta )-1]\times 100$$

where *β* is the slope of the log-linear regression for each segment. The average annual percent change (AAPC) summarizes the trend over the entire study period as a weighted average:

$$AAPC=[exp(\Sigma ({b}_{i}\times {w}_{i})/\Sigma {w}_{i})-1]\times 100$$

where *b*ᵢ is the slope coefficient for the *i*th segment and *w*ᵢ is the length of the *i*th segment. Model selection used the Bayesian Information Criterion (BIC) implemented in Joinpoint, Version 5.4.0.0; BIC penalises model complexity more strongly than the permutation test, providing a conservative selection criterion appropriate for short surveillance series with potentially correlated annual fluctuations [13]. We constrained the search to a maximum of two joinpoints per series (with a minimum of two observations between joinpoints, two observations at each end of the series, and a 12-year span), reflecting the maximum well-identified discontinuities supported by 12 annual observations and the well-documented programmatic and pandemic inflection points (End TB Strategy launch in 2015 and the COVID-19 disruption in 2020). To verify these settings we re-ran each region under nine alternative configurations crossing maximum joinpoints (1, 2, 3) with minimum-observations-per-segment of 2, 3 and 4. The BIC-optimal number of joinpoints was identical to the primary analysis in 11 of 13 regions and differed by only one for regions 2 and 13; estimated joinpoint years moved by ≤ 1 year in all sensitivity scenarios (Supplementary Table S3). Region-specific BIC values, joinpoint years and segment-level APC estimates with 95% *CI* are reported in Supplementary Table S3.

Generalized Additive Models (GAM) with cubic regression splines were fitted to the annual age-standardized TB incidence (ASR) for all 13 health regions using the *mgcv* package in R version 4.5.1 (2025-06-13 ucrt). The smoothing function was specified as s(Year, k = 5), where k = 5 represents the maximum allowable basis dimension; this value was selected via the gam.check diagnostic to balance flexibility against the 12-year sample size, and was supported by a k-index > 1 and effective degrees of freedom < 4 in every region. For each region, fitted smoothed values and pointwise 95% *CI* were extracted, and numerical first derivatives were computed via central differences to approximate the instantaneous annual percent change (APC).

$$APC=\left(\frac{d}{dt} SmoothASR/SmoothASR\right)\times 100$$

where *d/dt* is the first derivative with respect to time (year). Health region–specific average annual percent change (AAPC) was computed as the mean of the annual APC values across 2012–2023. GAM smoothing was applied to validate and cross-check the joinpoint-based estimates by providing a model-free assessment of long-term trend shape. Quantitative concordance was assessed using the coefficient of determination (*R*^2^) and mean absolute error (MAE) between observed and GAM-fitted ASR; across the 13 regions the mean *R*^2^ was 0.82 (range 0.35–0.97) and the mean MAE was 3.23 per 100,000 (range 0.81–5.04). The directional agreement between joinpoint and GAM AAPC was substantial: both methods classified 11 of 13 regions identically (declining vs non-declining), with the two discordances confined to regions 7 and 12, where the GAM derivative is sensitive to the early-period outliers that produce the joinpoint biphasic structure (Supplementary Table S4).

### Percent change analysis for classification

Provincial classification utilized percent change calculations defined as:

$$Percent Change=\left[\left({Rate}_{2}-{Rate}_{1}\right)/{Rate}_{1}\right]\times 100$$

Long-term classification (2012–2023): Provinces were classified using both the magnitude (effect size) and statistical reliability of the AAPC, addressing the well-documented limitation of *P*-value–only classification. Five categories were defined: (i) Strong decrease (AAPC ≤ − 5%, *P* < 0.05); (ii) Moderate decrease (− 5% < AAPC < 0%, *P* < 0.05); (iii) Stable or Unchanged (AAPC < 5% and *P* ≥ 0.05); (iv) Moderate increase (0% ≤ AAPC < 5%, *P* < 0.05); and (v) Strong increase (AAPC ≥ 5%, *P* < 0.05). Provinces with non-significant AAPC but AAPC ≥ 5% were flagged as “trend uncertain (declining)” or “trend uncertain (rising)” to highlight clinically important effect sizes that did not reach statistical significance because of limited series length. For continuity with the broader policy literature, we also retained the binary Safe Zone/Alarming Zone classification used in previous national TB surveillance reports (Safe Zone: AAPC < 0 with *P* < 0.05 OR *P* ≥ 0.05; Alarming Zone: AAPC ≥ 0 with *P* < 0.05) and present both classifications side-by-side in the Results.

COVID-19 impact assessment (2019–2023): To complement the long-term joinpoint analysis we examined short-term pandemic impact using both crude rate and age-standardized rate (ASR) percent change between 2019 (immediate pre-pandemic baseline) and 2023 (post-pandemic recovery year). The categorical thresholds (Decreased < − 5%, Unchanged − 5% to + 5%, Increased > + 5%) were chosen because (a) ± 5% approximates the typical year-to-year stochastic variation in provincial ASR documented in the pre-pandemic period (median absolute year-on-year change 2012–2019 = 4.9%) and (b) it aligns with the 5% threshold adopted by the WHO Global TB Report for flagging notable national-level changes in notifications. We additionally re-classified provinces using ± 10% and ± 15% thresholds and using the ASR-based change as a sensitivity analysis (Supplementary Table S5). Because the COVID-19 impact comparison spans only 4 years, the contribution of population aging to crude-rate change is expected to be small; the ASR-adjusted analysis nonetheless confirmed that 60 of 77 provinces (78.0%) showed a decrease and 15 (19.5%) an increase in age-standardized terms, broadly consistent with the crude-rate analysis.

### Spatial autocorrelation analysis: Moran’s* I* and LISA

Spatial autocorrelation was assessed using Global Moran’s *I* statistic [11, 20]:

$$Global Moran{\prime}s I=(n/{\Sigma}_{\mathrm{i}}{\Sigma}_{\mathrm{j}} {w}_{ij})\times [{\Sigma}_{\mathrm{i}} {\Sigma}_{\mathrm{j}} {w}_{ij}({x}_{i}-\overline{x })({x}_{j}-\overline{x })/{\Sigma}_{\mathrm{i}}{\left({x}_{i}-\overline{x }\right)}^{2}]$$

where *n* is the number of provinces, *x*ᵢ and *x*ⱼ are AAPC values for provinces *i* and *j*, *x̄* is the mean AAPC, and *w*ᵢⱼ is the spatial weight between provinces *i* and *j*. Values range from − 1 (perfect dispersion) to + 1 (perfect clustering), with 0 indicating random spatial distribution.

Local spatial clustering was identified using Local Indicators of Spatial Association (LISA) [11]:

$$Local LISA: {I}_{i}=({x}_{i}-\overline{x })\times {\Sigma}_{\mathrm{j}} {w}_{ij}({x}_{j}-\overline{x })/[{\Sigma}_{\mathrm{i}}{\left({x}_{i}-\overline{x }\right)}^{2}/n]$$

LISA identifies four types of spatial association: (1) High–High (HH): provinces with high AAPC surrounded by neighbors with high AAPC (hot spots); (2) Low–Low (LL): provinces with low AAPC surrounded by neighbors with low AAPC (cold spots); (3) High–Low (HL): high-AAPC provinces surrounded by low-AAPC neighbors (spatial outliers); (4) Low–High (LH): low-AAPC provinces surrounded by high-AAPC neighbors (spatial outliers).

Spatial analysis was performed using GeoDa 1.22.0.14 [21]. The primary spatial weights matrix used queen contiguity (shared boundary or vertex) with row standardisation, which was selected a priori because (a) Thailand’s administrative provinces share extensive land borders, (b) queen contiguity is the conventional first-order weighting scheme for province-level disease mapping in Southeast Asia, and (c) it is robust to the irregular polygon shapes typical of Thai provincial units. To assess robustness we re-computed Global Moran’s *I* and LISA cluster maps using three alternative weight specifications: rook contiguity, k-nearest-neighbour weights with k = 4, 6, and 8, and a 200-km distance band. To address concerns about multiple testing in the LISA framework we additionally applied the Benjamini–Hochberg false-discovery-rate (FDR) adjustment to the local Moran *P*-values at α = 0.05. To address the concern that aggregating temporal information into a single AAPC summary may attenuate true spatial structure, we additionally computed Global Moran’s *I* on the annual provincial ASR for each year 2012–2023 under each weight specification (Supplementary Table S6). Statistical significance was assessed using 999 permutations (*P* < 0.05).

### Software and statistical analysis

Statistical analyses were performed using: Joinpoint Regression Program, Version 5.4.0.0 (National Cancer Institute, Bethesda, MD, USA); GeoDa 1.22.0.14 (Center for Spatial Data Science, University of Chicago, Chicago, IL, USA) for spatial analysis; QGIS Desktop 3.40.2 (Open Source Geospatial Foundation, Beaverton, OR, USA) [22] for cartography; and R version 4.5.1 (2025-06-13 ucrt) (R Foundation for Statistical Computing, Vienna, Austria) [23] for data management and visualization. Maps were created using QGIS with provincial boundary shapefiles from the UN OCHA Thailand database. Statistical significance was set at α = 0.05 for all analyses, with *P* < 0.05 considered statistically significant.

## Results

### Overview of TB burden and national trends

Generalized Additive Model (GAM) analysis with 95% *CI* revealed substantial geographical heterogeneity in age-standardized tuberculosis incidence rates (ASR per 100,000 population) across Thailand’s 13 health regions during 2012–2023. The most dramatic declines were observed in health regions 3, 6, and 13, with observed ASR values decreasing from 41.87, 53.86, and 44.68 per 100,000 in 2012 to 2.83, 11.70, and 10.93 per 100,000 in 2023, respectively, representing reductions of 93.2%, 78.3%, and 75.5%. Health regions 4, 5, 9, and 11 demonstrated the strongest overall TB control performance, with observed ASR declining from 21.37, 25.19, 34.85, and 38.79 in 2012 to 2.11, 1.68, 2.16, and 2.94 per 100,000 in 2023, achieving reductions of 90.1%, 93.3%, 93.8%, and 92.4% respectively.

**Fig. 1:**
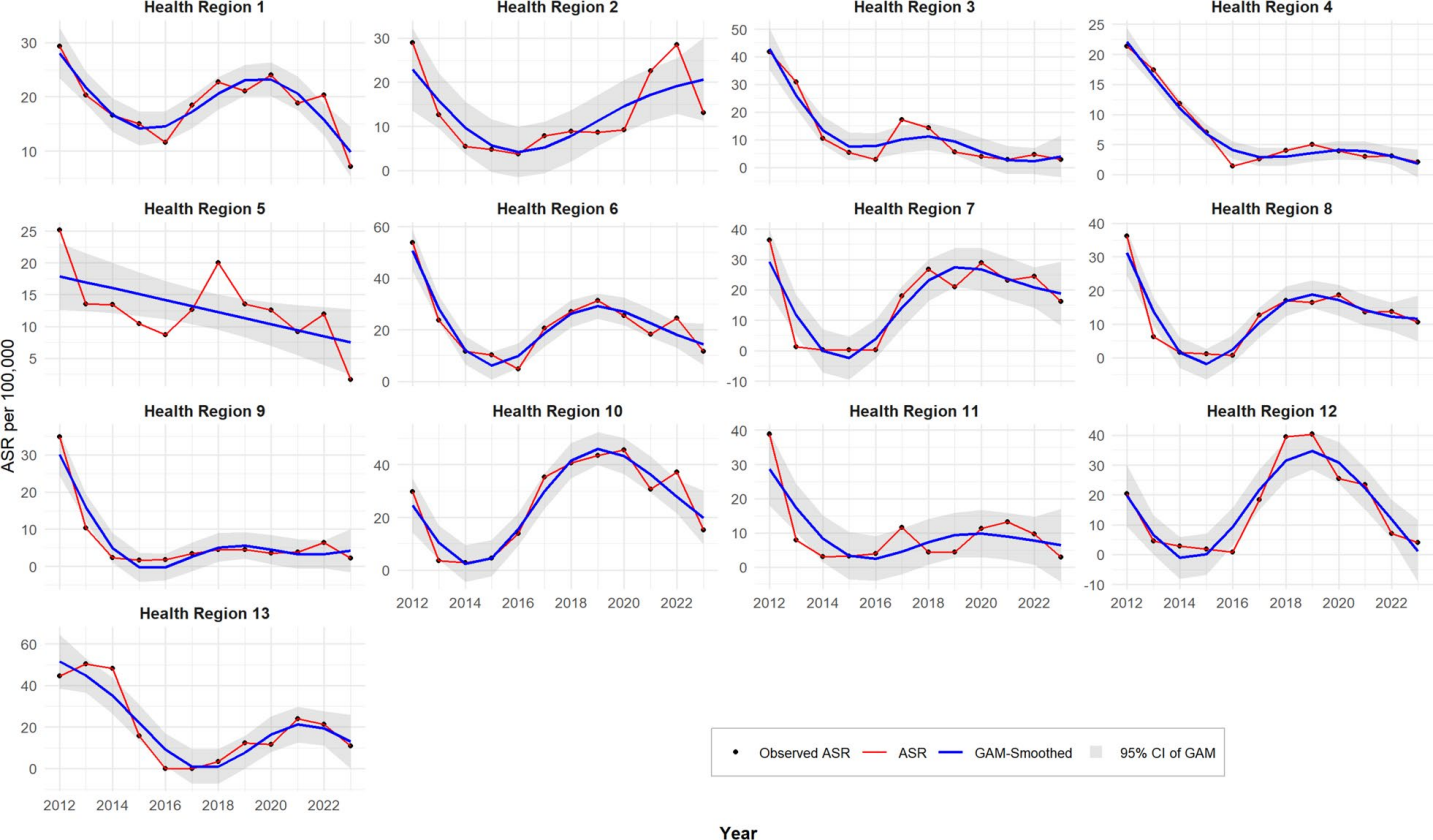
Age-standardized and GAM-smoothed trends of TB incidence rates for 13 health regions, 2012–2023

Health regions 4, 9, 11, and 5 demonstrated the strongest overall TB control performance, with observed ASR declining from 21.37, 34.85, 38.79, and 25.19 in 2012 to 2.11, 2.16, 2.94, and 1.68 per 100,000 in 2023, achieving reductions of 90.1%, 93.8%, 92.4%, and 93.3% respectively. Health regions 1, 7, and 8 showed substantial declines from baseline rates of 29.35, 36.46, and 36.16 to 7.11, 16.20, and 10.66 per 100,000, representing reductions of 75.8%, 55.6%, and 70.5%. Health region 2 exhibited a more moderate decline from 28.97 to 13.06 per 100,000 (54.9% reduction), while Health region 10 showed the smallest reduction from 29.70 to 15.24 per 100,000 (48.7% decline), and thus maintaining relatively higher incidence levels throughout the study period. Health region 12 displayed a distinctive biphasic pattern, initially declining from 20.34 to 4.06 per 100,000 (80.0% reduction overall) (Supplementary Table S1). The GAM-smoothed curves (GAM Fit values) demonstrated strong concordance with observed data across all regions, with narrow 95% *CI* indicating robust model performance and reliable trend estimation, validating the heterogeneous patterns of TB control success across Thailand’s diverse geographical and demographic contexts (Fig. 1).

### Regional temporal trends from joinpoint regression

The Joinpoint regression analysis revealed heterogeneous trends in TB incidence across Thailand’s 13 health regions during 2012–2023. Five health regions demonstrated statistically significant declining trends. Health region 3 exhibited the steepest decline with an average annual percent change (AAPC) of − 20.51% (95% *CI:* − 33.00 to − 13.77, *P* < 0.001), followed by health region 9 with an AAPC of − 19.42% (95% *CI:* − 28.98 to − 8.57, *P* = 0.003). Health region 4 showed a significant overall declining trend with an AAPC of − 17.53% (95% *CI:* − 23.64 to − 12.89, *P* < 0.001), characterized by two distinct segments: an initial sharp decline during 2012–2016 (APC: − 34.31%, 95% *CI:* − 51.65 to − 22.80, *P* < 0.001) followed by a more moderate, non-significant decline from 2016 to 2023 (APC: − 6.09%, 95% *CI:* − 18.36 to 45.13, *P* = 0.639). Additionally, a borderline significant decline was observed in health region 11 (AAPC = − 11.79%, 95% *CI:* − 22.16 to − 0.04; *P* = 0.049), whereas health region 13 exhibited a statistically significant decline with an AAPC of − 11.19% (95% *CI:* − 22.44 to − 3.74; *P* = 0.006).

Eight health regions exhibited non-significant trends over the study period. Health region 6 showed a biphasic pattern with an initial sharp decline during 2012–2014 (APC: − 46.12%, 95% *CI:* − 66.28 to − 4.06, *P* = 0.020) followed by a non-significant increase from 2014 to 2023 (APC: 6.21%, 95% *CI:* − 17.73 to 61.83, *P* = 0.202), resulting in an overall non-significant AAPC of − 6.12% (95% *CI:* − 13.21 to 3.20, *P* = 0.170). Similarly, health region 12 demonstrated a significant increase during 2012–2019 (APC: 20.20%, 95% *CI:* 4.42 to 96.66, *P* = 0.024) followed by a significant decline from 2019 to 2023 (APC: − 35.75%, 95% *CI:* − 94.89 to − 14.62, *P* = 0.020), yielding an overall non-significant AAPC of − 4.28% (95% *CI:* − 35.10 to 17.43, *P* = 0.468). Health regions 8 (AAPC: − 6.37%, 95% *CI:* − 15.49 to 3.73, *P* = 0.183), 5 (AAPC: − 6.05%, 95% *CI:* − 14.82 to 2.10, *P* = 0.124), 7 (AAPC: − 3.14%, 95% *CI:* − 13.32 to 13.66, *P* = 0.437), and 1 (AAPC: − 2.41%, 95% *CI:* − 9.08 to 4.66, *P* = 0.420) showed non-significant declining trends. Health region 2 (AAPC: 1.28%, 95% *CI:* − 8.92 to 12.63, *P* = 0.795) and health region 10 (AAPC: 4.91%, 95% *CI:* − 9.81 to 32.56, *P* = 0.420) exhibited non-significant increasing trends, with health region 10 showing the largest magnitude of increase among all regions (Table 1). We emphasise that the wide *CI* around the segment-specific APC estimates for the short post-2014 segment in region 6 (95% *CI* for APC 2014–2023: − 17.7% to + 61.8%) and the brief post-2019 segment in region 12 (95% *CI* for APC 2019–2023: − 94.9% to − 14.6%) reflect the small number of observations available within those segments. Sensitivity re-analyses with stricter minimum-segment lengths (3 and 4 observations) eliminated the second segment in region 6 and shortened the post-pandemic decline interval in region 12 by one year, but did not alter the direction of the temporal pattern; all consistency checks are reported in Supplementary Table S3. These segment-specific estimates should therefore be interpreted as broad indicators of trend reversal rather than precise rate-of-change estimates.

**Table 1:** Joinpoint regression analysis of tuberculosis incidence trends by health region, Thailand, 2012–2023

Region	Segment	Period	APC (%)	95% *CI*	*P* value	AAPC (%)	95% *CI*	*P* value
1	0	2012–2023	− 2.41	(− 9.08, 4.66)	0.420	− 2.41	(− 9.08, 4.66)	0.420
2	0	2012–2023	1.28	(− 8.92, 12.63)	0.795	1.28	(− 8.92, 12.63)	0.795
3	0	2012–2023	− 20.51*	(− 33.00, − 13.77)	< 0.001	− 20.51*	(− 33.00, − 13.77)	< 0.001
4	0	2012–2016	− 34.31*	(− 51.65, − 22.80)	< 0.001	− 17.53*	(− 23.64, − 12.89)	< 0.001
	1	2016–2023	− 6.09	(− 18.36, 45.13)	0.639			
5	0	2012–2023	− 6.05	(− 14.82, 2.10)	0.124	− 6.05	(− 14.82, 2.10)	0.124
6	0	2012–2014	− 46.12*	(− 66.28, − 4.06)	0.020	− 6.12	(− 13.21, 3.20)	0.170
	1	2014–2023	6.21	(− 17.73, 61.83)	0.202			
7	0	2012–2023	− 3.14	(− 13.32, 13.66)	0.437	− 3.14	(− 13.32, 13.66)	0.437
8	0	2012–2023	− 6.37	(− 15.49, 3.73)	0.183	− 6.37	(− 15.49, 3.73)	0.183
9	0	2012–2023	− 19.42*	(− 28.98, − 8.57)	0.003	− 19.42*	(− 28.98, − 8.57)	0.003
10	0	2012–2023	4.91	(− 9.81, 32.56)	0.420	4.91	(− 9.81, 32.56)	0.420
11	0	2012–2023	− 11.79*	(− 22.16, − 0.04)	0.049	− 11.79*	(− 22.16, − 0.04)	0.049
12	0	2012–2019	20.20*	(4.42, 96.66)	0.024	− 4.28	(− 35.10, 17.43)	0.468
	1	2019–2023	− 35.75*	(− 94.89, − 14.62)	0.020			
13	0	2012–2023	− 11.19*	(− 22.44, − 3.74)	0.006	− 11.19*	(− 22.44, − 3.74)	0.006

Note: *APC* annual percent change for each segment; *AAPC* average annual percent change for entire period (2012–2023); *CI* confidence interval; * statistically significant at α = 0.05 level (two-tailed test)

### Spatial autocorrelation analysis

**Fig. 2:**
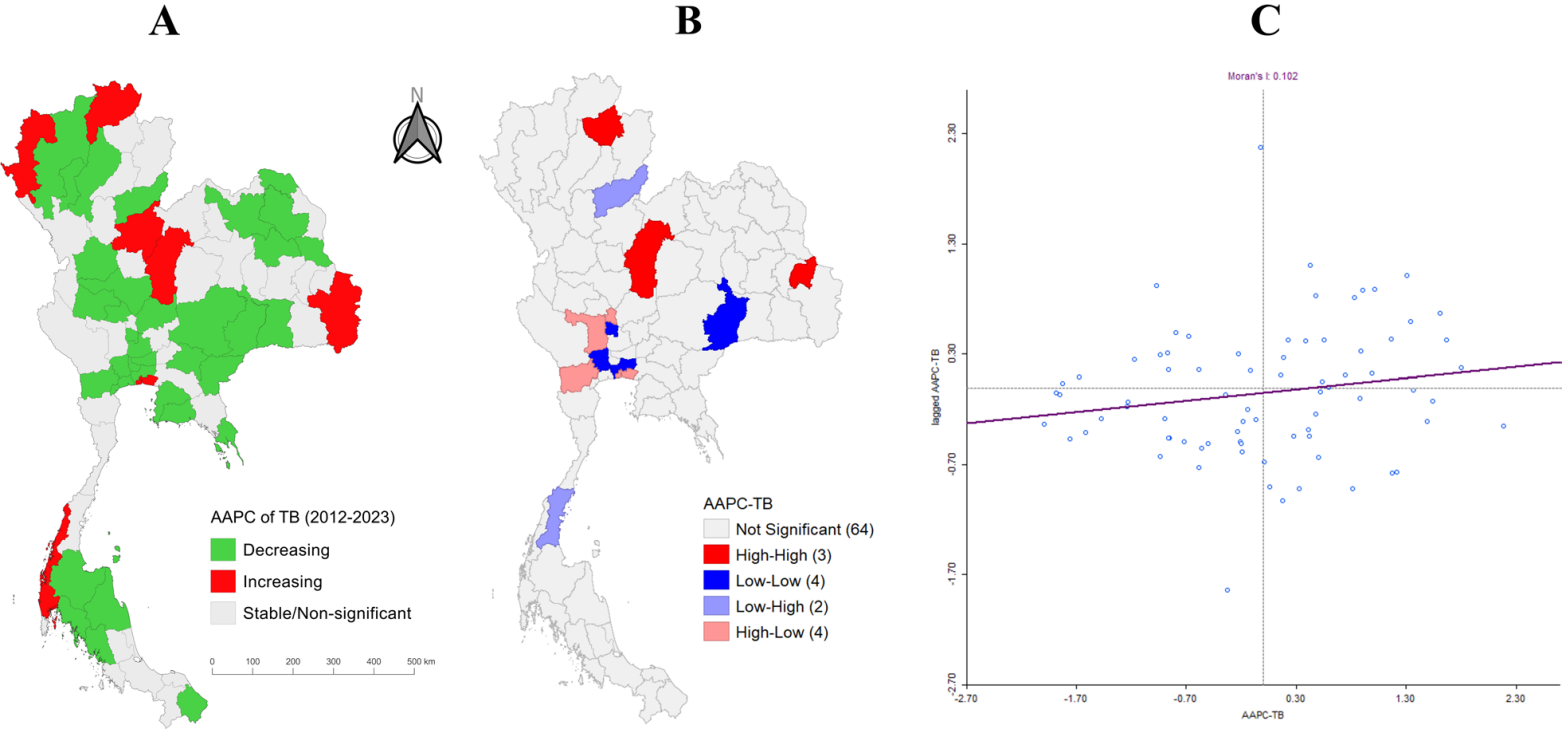
Spatial analysis results: **A** provincial AAPC choropleth map; **B** LISA cluster map; **C** Moran scatterplot. *AAPC* Average annual percent change; *LISA* Local Indicators of Spatial Association; *TB* Tuberculosis

Global Moran’s *I* of provincial AAPC values indicated weak spatial autocorrelation (*I* = 0.102, *P* = 0.058 under queen contiguity; 999 permutations), suggesting only modest geographic structuring of long-term TB trends. Sensitivity analyses using rook contiguity (*I* = 0.097, *P* = 0.072), k-nearest-neighbour weights (k = 4: *I* = 0.009; k = 6: *I* = 0.051; k = 8: *I* = 0.005), and a 200-km distance band (*I* = − 0.020) produced consistent conclusions of weak global clustering, confirming that the result is not an artefact of the queen-contiguity specification. Year-by-year Global Moran’s *I* computed on annual provincial ASR ranged from − 0.114 to + 0.094 across 2012–2023 with no individual year significant at *P* < 0.05 after Benjamini–Hochberg FDR correction (Supplementary Table S6), reinforcing the conclusion that province-specific factors rather than spatial diffusion dominate Thailand’s TB epidemiology. After FDR correction at the 5% level, eight of the original 13 LISA-significant provinces remained significant: the three High–High hot-spots (Phayao, Phetchabun, Yasothon), the four Low–Low cold-spots (Buri Ram, Ang Thong, Nakhon Pathom, Bangkok) and one High–Low outlier (Samut Prakan); the four borderline outliers (Suphan Buri, Sing Buri, Ratchaburi, Uttaradit, Chumphon) lost significance after FDR adjustment and are interpreted with caution (Fig. 2C).

Local Indicators of Spatial Association (LISA) identified 13 provinces (16.9%) with significant local spatial autocorrelation, categorized into four types (Fig. 2B). Hot-spots, cold-spots and spatial outliers are described below.

High–High (HH) clusters—Hot spots (3 provinces): Phayao (AAPC: + 8.21%), Phetchabun (AAPC: + 5.37%), Yasothon (AAPC: + 4.92%). These provinces and their neighbors exhibited persistently high or increasing TB rates, indicating the need for priority intervention.

Low–Low (LL) clusters—Cold spots (4 provinces): Buri Ram (AAPC: − 25.70%), Ang Thong (AAPC: − 30.33%), Nakhon Pathom (AAPC: − 38.25%), Bangkok (AAPC: − 20.05%). These provinces demonstrated sustained TB control success with declining rates, representing potential models for best practice dissemination.

High–Low (HL) outliers (4 provinces): Suphan Buri (AAPC: + 9.97%), Sing Buri (AAPC: + 8.15%), Ratchaburi (AAPC: + 6.82%), Samut Prakan (AAPC: + 7.35%). These provinces showed increasing trends despite the surrounding provinces with declining trends.

Low–High (LH) outliers (2 provinces): Uttaradit (AAPC: − 16.47%), Chumphon (AAPC: − 18.92%).

### Provincial long-term classification (2012–2023)

**Fig. 3:**
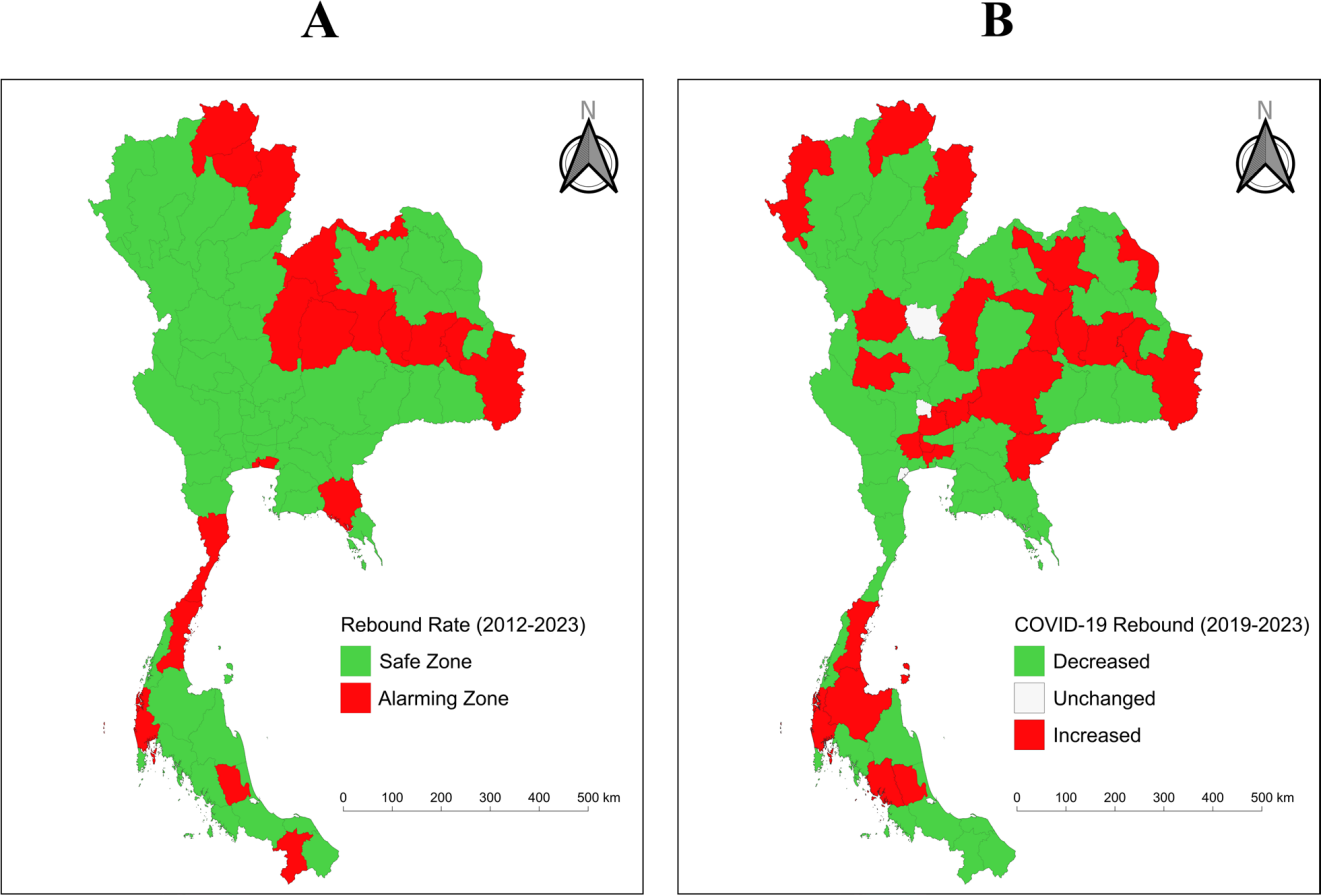
Spatial comparison maps: **A** long-term classification (2012–2023)—choropleth of average annual percent change (AAPC) by province; green shades indicate decreases (Safe Zone) and red shades indicate increases (Alarming Zone); **B** COVID-19 period change (2019–2023)—choropleth of AAPC differences showing increased (red), decreased (green) and unchanged (gray) provinces

Provincial-level joinpoint analysis yielded a long-term classification of 77 provinces based on AAPC values and statistical significance (Fig. 3A):

Under the long-standing binary scheme, 69 provinces (89.6%) fell into the Safe Zone (decreasing or stable AAPC) and 8 provinces (10.4%) into the Alarming Zone (significant increasing AAPC); the Safe Zone comprised 36 provinces (46.8%) with significant decreasing trends (AAPC < 0, *P* < 0.05) and 33 provinces (42.9%) with non-significant trends (*P* ≥ 0.05). Applying the new five-category effect-size–and–significance classification revealed clinically important heterogeneity within the Safe Zone: 36 provinces (46.8%) demonstrated a Strong decrease (AAPC ≤ − 5%, *P* < 0.05); 17 provinces (22.1%) were genuinely Stable (AAPC < 5%, *P* ≥ 0.05); and 16 provinces (20.8%) showed “trend uncertain” patterns with AAPC ≥ 5% but wide *CI*—seven trending downward (potential late-stage progress) and nine trending upward (potential emerging risk that the binary scheme had labelled “safe”). These nine “trend uncertain (rising)” provinces include Sing Buri, Songkhla, Roi Et, Yasothon, Chaiyaphum, Phuket, Loei, Nong Bua Lam Phu, and Phatthalung, which warrant intensified surveillance even though their AAPCs did not reach the conventional *P* < 0.05 threshold (Supplementary Table S5).

**Alarming Zone (8 provinces, 10.4%):** Provinces with significant increasing trends (AAPC ≥ 0, *P* < 0.05). These provinces are: Mae Hong Son, Phayao, Phetchabun, Suphan Buri, Sing Buri, Ratchaburi, Samut Prakan, and Yasothon. Geographically, these provinces span the Northern, Central, Northeastern, and Western regions of the country.

### COVID-19 pandemic impact assessment (2019–2023)

Comparison of 2023 vs 2019 baseline crude rates revealed heterogeneous pandemic impact across provinces (Table 2, Fig. 3B):

- **Decreased (49 provinces, 63.6%):** Provinces showing > 5% decline in crude rates. These provinces represent 56.4% of the national population but only 37.2% of the 2023 TB case burden, with a mean crude rate of 10.13 per 100,000.
- **Unchanged (3 provinces, 3.9%):** Provinces with changes between − 5% and + 5%, indicating stable rates during the COVID-19 period.
- **Increased (25 provinces, 32.5%):** Provinces showing > 5% increase in crude rates. Despite representing only 42.5% of the national population, these provinces accounted for 62.6% of the 2023 TB case burden, with a mean crude rate of 23.65 per 100,000. ASR-based sensitivity analysis using age-standardized rates between 2019 and 2023 (Supplementary Table S5) identified 60 provinces (77.9%) as Decreased, 2 (2.6%) as Unchanged and 15 (19.5%) as Increased—a directionally consistent but more conservative estimate of pandemic disruption. Concordance between the crude-rate and ASR-based classifications was 70.1%; the 23 provinces reclassified moved predominantly from “Increased” (crude) to “Decreased” (ASR), suggesting that part of the apparent COVID-related rise in crude TB notifications reflected demographic ageing rather than true incidence increase. Re-classifying using ± 10% and ± 15% ASR thresholds (Supplementary Table S5) yielded 14 and 13 provinces, respectively, in the Increased category—a stable subset of provinces (Mae Hong Son, Bangkok, Sing Buri, Roi Et, Yasothon, Phichit, Phetchabun, Ratchaburi, Samut Prakan and five others) appears in every sensitivity scenario and represents the highest-priority pandemic-recovery targets.

**Table 2:** COVID-19 pandemic impact on provincial TB rates (2019 vs. 2023 comparison)

Change category	Provinces (*n*, %)	Population 2023 (*n*, %)	TB cases 2023 (*n*, %)	Mean crude rate (per 100,000)
Decreased	49 (63.6)	38,725,944 (56.4)	3923 (37.2)	10.13
Unchanged	3 (3.9)	944,049 (1.4)	18 (0.2)	1.91
Increased	25 (32.5)	29,216,305 (42.5)	6563 (62.6)	23.65
TOTAL	77 (100)	68,886,298 (100)	10,486 (100)	15.27

Note: *n* number; *TB* Tuberculosis

## Discussion

This nationwide spatiotemporal analysis (2012–2023) yields three principal findings of policy relevance. First, although national TB incidence declined substantially over the decade, regional heterogeneity is pronounced: five of the 13 health regions achieved statistically significant long-term declines, two displayed biphasic trajectories suggestive of programmatic or surveillance transitions, and several others showed slower, non-significant change. Second, provincial-level spatial analysis identified only weak global clustering but locally meaningful structure, with High–High hot-spots, Low–Low cold-spots and spatial outliers that delineate priority intervention and best-practice districts. Third, the 2019–2023 pandemic period produced a geographically concentrated burden disruption, with a minority of provinces accounting for a disproportionate share of the 2023 case load. Together these findings argue against uniform national responses and in favour of geographically targeted, equity-oriented control measures.

The magnitude of tuberculosis burden reduction observed in Thailand’s high-performing regions substantially exceeds that documented in comparable settings globally. Regional joinpoint analyses from China reported AAPC values ranging from − 3.8% to − 8.2% during 2005–2016, with no regions approaching double-digit annual declines [24]. Similarly, India’s district-level joinpoint analysis documented AAPC values between − 2.1% and − 6.5% for the period 2001–2014, with the highest-performing districts achieving approximately 5–7% annual reductions [25]. Other high-burden settings in Southeast Asia have demonstrated more gradual declining trends, typically in the range of 3–6% annual reduction [26]. Thailand’s achievement of 10–20% annual declines (AAPC) in five regions thus represents exceptional tuberculosis control performance, warranting detailed programmatic analysis to identify replicable intervention strategies which could inform accelerated progress toward elimination goals in similar epidemiological contexts.

The biphasic patterns observed in regions 6 and 12 merit careful interpretation within the context of surveillance system evolution and programmatic transitions. Region 6’s initial sharp decline (APC: − 46.12% during 2012–2014) followed by stabilization resembles patterns documented in settings implementing intensive short-term interventions, such as enhanced active case-finding campaigns or targeted screening programs, which produce rapid initial gains followed by plateau effects as intervention intensity normalizes or case detection reaches saturation among high-risk populations. Region 12’s mid-period increase (APC: + 20.20% during 2012–2019) followed by a subsequent sharp decline (APC: − 35.75% during 2019–2023) coincides temporally with the WHO End TB Strategy launch in 2015 [4], suggesting a possible programmatic transition affecting case detection sensitivity, diagnostic technology adoption, or reporting practices. Similar mid-period fluctuations have been documented in settings undergoing diagnostic system modernization, particularly with the implementation of genomic rapid diagnostics (Xpert MTB/RIF) that can temporarily increase case notifications through improved diagnostic sensitivity and reduced time to detection before stabilizing as the technology becomes integrated into routine practice [27]. Three plausible explanations warrant consideration for these temporal patterns: first, changes in surveillance system sensitivity or case definitions during the mid-2010s may have produced artifactual volatility rather than reflecting genuine epidemiological dynamics; second, genuine programmatic interventions may have generated substantial short-term effects on case detection and notification; third, intensified active case-finding campaigns may have temporarily increased notifications by identifying previously undetected prevalent cases before settling to lower incidence levels reflecting reduced transmission.

The observed spatial autocorrelation (Moran’s *I* = 0.102) demonstrates only weak spatial structuring, markedly lower than that reported in Ethiopia (*I* = 0.291) [28] and Pakistan (*I* = 0.185) [29], suggesting that TB patterns in Thailand are shaped by modest spatial influences alongside province-specific determinants. LISA-identified High–High clusters (Phayao, Phetchabun, Yasothon) represent priority areas requiring intensive interventions targeting potential contributing factors including border proximity facilitating cross-border transmission [30, 31], high poverty levels reducing access to healthcare [32, 33], migrant populations with limited access [34], and HIV co-infection requiring integrated services [30]. Conversely, Low–Low clusters (Buri Ram, Ang Thong, Nakhon Pathom, Bangkok) demonstrating sustained success warrant investigation to identify transferable best practices. Bangkok’s achievement despite high population density suggests that effective urban strategies are in place that could inform interventions elsewhere. Detailed programmatic assessment of these provinces should examine intervention approaches, healthcare delivery models, community engagement initiatives, and digital health applications to identify evidence-based approaches that could be disseminated to surrounding and demographically similar areas. High–Low outliers (Suphan Buri, Sing Buri, Ratchaburi, Samut Prakan) showing increasing trends despite declining neighbors merit urgent investigation of local drivers, including industrial development attracting migrants, population influx concentrating vulnerable groups, healthcare capacity constraints, drug-resistant strain emergence, or programmatic breakdowns requiring tailored corrective interventions.

The provincial classification confirms that the great majority of Thai provinces have moved into or remained within a low-burden trajectory, while a small but coherent set of Alarming-Zone and “trend uncertain (rising)” provinces concentrate the residual risk. The 2019–2023 pandemic comparison reinforces this pattern: a minority of provinces account for a disproportionate share of the 2023 case load, and overlap between these provinces and the long-term Alarming Zone defines a compound-vulnerability subset that should be prioritised for combined pandemic-recovery and programmatic-strengthening action. The observed concentration is consistent with the global WHO finding of 25–40% notification declines during the pandemic [35–37] and with the disproportionate burden borne by vulnerable populations [5, 6, 38]. Recovery strategies should therefore combine intensified active case-finding [39], integrated TB–COVID-19 services leveraging strengthened respiratory infrastructure [40], community-based continuity-of-care models [41], and digital-health remote monitoring. The first joinpoint inflection (2014–2016) clustered around the launch of the WHO End TB Strategy (2015) and Thailand’s adoption of the National Strategic Plan for Tuberculosis (2017–2021), which expanded Xpert MTB/RIF deployment and active case-finding among high-risk groups; the second inflection (2019–2021) coincided with COVID-19–related interruptions to TB diagnostic services and supervised treatment. These programmatic links should be considered hypothesis-generating and warrant dedicated evaluation studies.

This study’s primary strength lies in comprehensive national surveillance data spanning 77 provinces over 12 years (2012–2023), enabling robust spatiotemporal analysis. Methodological rigor through joinpoint regression identifying significant trend changes, generalized additive models with 95% *CI*, spatial autocorrelation analysis (Moran’s *I*), and Local Indicators of Spatial Association provides triangulated evidence of heterogeneous tuberculosis dynamics. Age-standardized incidence rates ensure temporal and geographic comparability, while an extended time period captures both long-term trends and COVID-19 pandemic impacts. However, several important limitations warrant consideration. As an ecological study using aggregate provincial data, findings cannot establish individual-level causality or directly assess individual risk factors. The biphasic patterns in regions 6 and 12, while statistically significant, require further investigation to distinguish programmatic or surveillance changes from genuine epidemiological dynamics. The Exact Method applied for zero-case observations introduced some statistical uncertainty, although sensitivity analysis confirmed that the overall result remained robust. Absence of linked data on treatment outcomes, drug resistance prevalence, and HIV co-infection status limits comprehensive burden assessment and prevents evaluation of program quality indicators beyond case notification. Spatial analyses using administrative boundaries may not reflect actual transmission networks or healthcare catchment areas. Finally, distinguishing between real changes in transmission and case detection sensitivity variations remains challenging without genomic epidemiological data. We additionally acknowledge several limitations highlighted by external review. First, the analytical design intentionally focused on regional and provincial heterogeneity to inform geographically-targeted policy; sex- and age-stratified provincial trend analyses were not undertaken because the routine Report 506 extracts available to the research team did not include record-level sex stratification at the provincial-by-year resolution required for joinpoint analysis without compromising small-cell confidentiality. We acknowledge that TB epidemiology differs by sex and across the life course, and future work using individual-level case data including sex-disaggregated and age-cohort analyses, as well as integration of HIV co-infection and drug-resistance status, represents an important extension of the present geographic framework. Second, the segment-specific APC estimates for short post-joinpoint intervals (region 6 post-2014 and region 12 post-2019) carry wide *CI*; we have expanded their interpretation as indicators of trend direction rather than precise rate estimates and have triangulated them with GAM-derived smoothed trajectories. Third, while the + 0.5 zero-case correction had negligible impact on AAPC estimates (Supplementary Table S2), residual underreporting during the COVID-19 disruption cannot be excluded; we have therefore reported the ASR-based COVID sensitivity analysis (Supplementary Table S5) and we encourage cautious interpretation of 2020–2022 estimates. Fourth, the queen-contiguity weights are robust to alternative spatial weight specifications and to FDR multiple-testing correction, but, as for any administrative-boundary–based analysis, the resulting LISA clusters may not coincide with actual transmission catchments and should be considered epidemiological signal rather than transmission-tree inference.

## Conclusion and policy implications

Findings support five priority policy actions for Thailand’s tuberculosis control program. First, implement intensive intervention packages in eight alarming zone provinces (Mae Hong Son, Suphan Buri, Sing Buri, Ratchaburi, Samut Prakan, Phayao, Phetchabun, Yasothon) and three High–High clusters (Phayao, Phetchabun, Yasothon), prioritizing enhanced active case-finding, strengthened treatment support, and comprehensive contact investigation. Second, conduct detailed programmatic assessments of four Low–Low cluster provinces (Buri Ram, Ang Thong, Nakhon Pathom, Bangkok) to identify and disseminate successful strategies as evidence-based intervention packages for adaptation in underperforming areas. Third, prioritize pandemic recovery efforts in 25 provinces with increased tuberculosis burden during 2019–2023, ensuring strengthened surveillance, diagnostic capacity, and service delivery continuity. Fourth, investigate mechanisms underlying biphasic patterns in regions 6 and 12 through a detailed review of programmatic changes, surveillance modifications, and diagnostic technology adoption during inflection periods. Fifth, address compound vulnerability in provinces demonstrating both long-term deterioration and pandemic-related disruption through multi-sectoral approaches targeting social determinants.

Future research priorities include: validation studies assessing surveillance data quality and completeness; qualitative investigations of successful control strategies in high-performing provinces; multilevel modeling incorporating individual, community, and programmatic factors; genomic epidemiological studies clarifying transmission dynamics during volatile periods; and prospective monitoring of pandemic recovery trajectories with evaluation of catch-up strategy effectiveness. Recommendations should be interpreted in light of the study’s ecological design and the data limitations described above. In particular, the eight Alarming-Zone provinces and the additional “trend uncertain (rising)” provinces identified by the new effect-size classification (Supplementary Table S5) together define a coherent set of priority districts where provincial public health offices could pilot enhanced active case-finding linked to migrant-worker outreach, integrated TB-HIV-diabetes screening, and digital adherence support. Sustained monitoring through annual updates to this spatiotemporal framework, combined with future sex- and age-disaggregated and resistance-stratified analyses, will be essential to verify whether the post-2019 trajectories represent durable recovery or transient catch-up in TB notifications.

## Supplementary Information

Additional file 1: Figure S1: Joinpoint regression trends and APC values across the 13 health regions, 2012–2023

Additional file 2

Additional file 3: Table S1. Annual age-standardized tuberculosis incidence ratesfor Thailand’s 13 health regions, standardized to the WHO World Standard Population. Table S2. Sensitivity analysis of zero-case + 0.5 correction: comparison of regional AAPC estimates under four zero-handling methods. Table S3. Joinpoint sensitivity analysis: BIC-optimal joinpoint number and locations for all 13 health regions across nine combinations of maximum-joinpointsand minimum-observations-per-segment. Table S4. Quantitative concordance between joinpoint and GAM-derived AAPC by health region. Table S5. Effect-size–and–significance classification of all 77 provinces and ASR-based COVID-19 impact sensitivity analysis at ± 5%, ± 10% and ± 15% thresholds. Table S6. Annual Global Moran’s I for provincial ASRunder queen, rook, k-nearest-neighbourand 200-km distance-band weights, with Benjamini–Hochberg FDR-adjusted P-values. Table S7. List of the 13 LISA-significant provinces with FDR-adjusted local Moran *P*-values and cluster classification.

## Data Availability

TB surveillance data are available from the Bureau of Epidemiology, Department of Disease Control, Ministry of Public Health Thailand (https://ddc.moph.go.th). Population data are publicly available from the National Statistical Office Thailand (http://www.nso.go.th). The datasets used and/or analyzed in this study are accessible from the corresponding author upon reasonable request.

## Abbreviations

AAPC: Average annual percent change
APC: Annual percent change
ASR: Age-standardized rate
BIC: Bayesian information criterion
*CI*: Confidence interval
GAM: Generalized additive model
HH: High–High (spatial cluster)
HL: High–Low (spatial outlier)
LH: Low–High (spatial outlier)
LISA: Local Indicators of Spatial Association
LL: Low–Low (spatial cluster)
SE: Standard error
TB: Tuberculosis
WHO: World Health Organization
