# Supplementary figures and images for: Spatiotemporal trends in tuberculosis incidence in Thailand, 2012–2023: a nationwide, province-level analysis
Source: Infect Dis Poverty. 2026 Jul 1;15:72. doi: 10.1186/s40249-026-01473-2 (PMC13321431; doi:10.1186/s40249-026-01473-2)

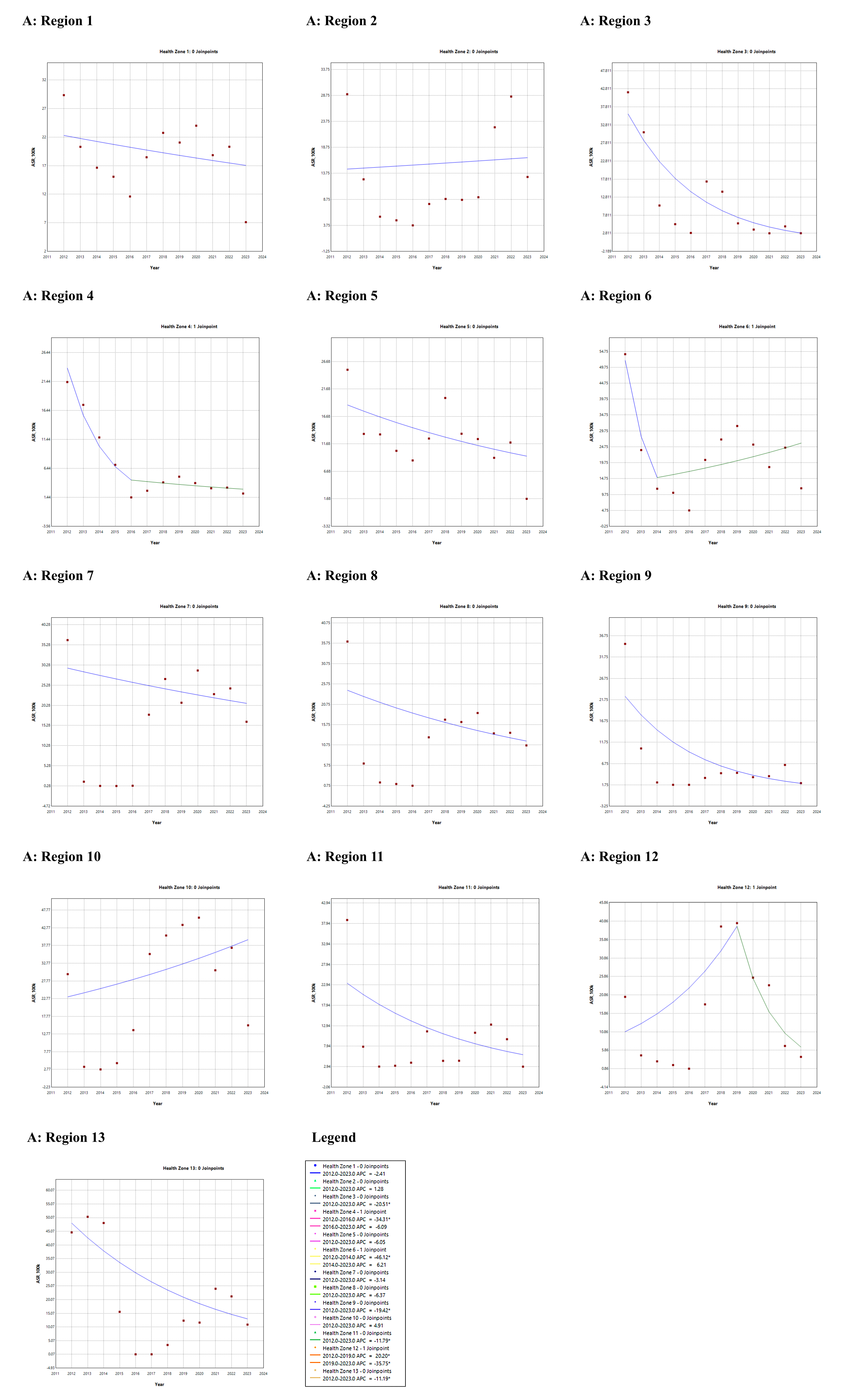

Supplement: Supplementary file 1 — Additional file 1: Figure S1: Joinpoint regression trends and APC values across the 13 health regions, 2012–2023 [file 40249_2026_1473_MOESM1_ESM.png]
