# Supplementary material for: Spatiotemporal trends in tuberculosis incidence in Thailand, 2012–2023: a nationwide, province-level analysis
Source: Infect Dis Poverty. 2026 Jul 1;15:72. doi: 10.1186/s40249-026-01473-2 (PMC13321431; doi:10.1186/s40249-026-01473-2)
Supplement: Supplementary file 2 — Additional file 2 [file 40249_2026_1473_MOESM2_ESM.docx]

**Supplementary materials**

*Spatiotemporal trends in tuberculosis incidence in Thailand, 2012–2023: a nationwide, province-level analysis*

# **Supplementary Note S1. List of 77 provinces grouped by 13 health regions**

Thailand’s Ministry of Public Health (MOPH) organises the country’s 77 provinces into 13 health service regions (zones). Each region operates as an administrative health service network coordinated by a regional health office. The province–region assignment used throughout this analysis follows the official MOPH Health Service Region classification, which is summarised in **Supplementary Table S0** below.

**Supplementary Table S0.** Thailand’s 77 provinces grouped by 13 health service regions (Ministry of Public Health classification).

| **Health Region** | **Geographic zone** | **Provinces** | **n** |
| --- | --- | --- | --- |
| 1 | Northern Thailand (upper) | Chiang Mai, Mae Hong Son, Lampang, Lamphun, Chiang Rai, Nan, Phayao, Phrae | 8 |
| 2 | Northern Thailand (lower) | Tak, Phitsanulok, Phetchabun, Sukhothai, Uttaradit | 5 |
| 3 | Central Thailand (upper) | Kamphaeng Phet, Nakhon Sawan, Phichit, Uthai Thani, Chai Nat | 5 |
| 4 | Central Thailand (Bangkok periphery) | Nonthaburi, Pathum Thani, Phra Nakhon Si Ayutthaya, Saraburi, Lopburi, Sing Buri, Ang Thong, Nakhon Nayok | 8 |
| 5 | Western Thailand | Kanchanaburi, Nakhon Pathom, Ratchaburi, Suphan Buri, Prachuap Khiri Khan, Phetchaburi, Samut Sakhon, Samut Songkhram | 8 |
| 6 | Eastern Thailand | Chonburi, Rayong, Chanthaburi, Trat, Samut Prakan, Chachoengsao, Prachinburi, Sa Kaeo | 8 |
| 7 | Northeastern Thailand (central) | Khon Kaen, Maha Sarakham, Roi Et, Kalasin | 4 |
| 8 | Northeastern Thailand (upper) | Udon Thani, Nong Khai, Bueng Kan, Nong Bua Lamphu, Loei, Sakon Nakhon, Nakhon Phanom | 7 |
| 9 | Northeastern Thailand (lower, west) | Nakhon Ratchasima, Buriram, Surin, Chaiyaphum | 4 |
| 10 | Northeastern Thailand (lower, east) | Ubon Ratchathani, Sisaket, Yasothon, Amnat Charoen, Mukdahan | 5 |
| 11 | Southern Thailand (upper, Andaman/Gulf) | Chumphon, Surat Thani, Nakhon Si Thammarat, Ranong, Phang Nga, Phuket, Krabi | 7 |
| 12 | Southern Thailand (lower, Deep South) | Phatthalung, Trang, Songkhla, Satun, Pattani, Yala, Narathiwat | 7 |
| 13 | Bangkok Metropolis | Bangkok | 1 |
| **Total** | |  | **77** |

Note: Province names are reported in the Royal Thai General System of Transcription. The 13-region classification has been the official MOPH structure since 2012; province–region assignments are stable across the study period (2012–2023). Bangkok constitutes a single-province metropolitan health region (Region 13) administered separately from the 12 provincial health regions.

# **Supplementary Note S2. National Disease Surveillance System (Report 506): data source, access conditions and license**

## **Data source and scope**

The TB case notification data analysed in this study were extracted from the National Disease Surveillance System (“Report 506”; Thai: รายงาน 506), the routine notifiable-disease surveillance platform operated by the Bureau of Epidemiology, Department of Disease Control, Ministry of Public Health, Thailand (https://apps-doe.moph.go.th/boeeng/annual.php). Report 506 has been Thailand’s national notifiable-disease reporting system since 1991 and captures monthly case notifications for tuberculosis and 76 other notifiable communicable diseases, reported by every district hospital, regional hospital and registered private healthcare facility nationwide. Tuberculosis is classified as a Group 3 notifiable disease (“diseases of public health importance”) under the Communicable Diseases Act B.E. 2558 (2015), and reporting is mandatory for all healthcare providers.

## **Data access and licence conditions**

Two access tiers exist for Report 506 data:

**(i) Public aggregate data.** Annual provincial- and national-level aggregate case counts by ICD-10 code, sex band and 5-year age band are openly available without registration at the Bureau of Epidemiology Annual Surveillance Reports portal (https://apps-doe.moph.go.th/boeeng/annual.php) and the Disease Data Center (DDC) open-data platform (https://ddc.moph.go.th). These public aggregate data were the primary input for the present analysis. No individual identifiers are present in the public release. The public aggregate data are released under the Government of Thailand Open Data Licence (compatible with CC BY 4.0), permitting re-use for research, education and public communication provided the source is cited as: “Bureau of Epidemiology, Department of Disease Control, Ministry of Public Health, Thailand (National Disease Surveillance System, Report 506).”

**(ii) Record-level (de-identified) data.** Record-level extracts (one row per notification, with sex, age in years, diagnosis date, residence district, ICD-10 code, treatment outcome) are restricted-access and require: (a) a formal written data-request to the Director, Bureau of Epidemiology, Department of Disease Control, Ministry of Public Health (“dcd@health.moph.go.th”); (b) institutional research ethics approval from a Thai Ethics Review Committee that has reciprocal agreement with the MOPH; (c) a signed Data Use Agreement (DUA) prohibiting record linkage, re-identification and onward sharing; and (d) where applicable, a project-specific Memorandum of Understanding between the requesting institution and the Department of Disease Control. Approval is normally granted within 30–60 working days. Record-level data were NOT used in the present analysis; all results in this manuscript derive exclusively from the public aggregate tier.

## **Ethics statement**

Because the analysis used only aggregate provincial counts already in the public domain, the Khon Kaen University Ethics Committee for Human Research issued a formal exemption from full ethical review (reference HE 682254). The exemption is consistent with the Council for International Organizations of Medical Sciences (CIOMS) International Ethical Guidelines for Health-related Research Involving Humans (2016), Guideline 12 (Collection, storage and use of data in health-related research), which exempts secondary analysis of fully de-identified, publicly released aggregate surveillance data from requirements for individual informed consent.

## **Suggested data-source citation**

Bureau of Epidemiology, Department of Disease Control, Ministry of Public Health, Thailand. National Disease Surveillance System (Report 506) – Tuberculosis annual notifications by province, 2012–2023. Nonthaburi: Bureau of Epidemiology; 2024. Available from: https://apps-doe.moph.go.th/boeeng/annual.php (accessed 15 December 2025).

# **Supplementary Figure S1**

**Supplementary Figure S1.** Joinpoint regression trends and segment-specific annual percent change (APC) values across the 13 health regions of Thailand, 2012–2023. Panels A–M correspond to health regions 1–13 respectively; each panel plots observed age-standardized TB incidence (dots), Joinpoint regression fit (solid line) and the locations of statistically identified joinpoints. Segment-specific APC values are annotated; an asterisk (*) denotes statistical significance at α = 0.05 (two-tailed). This figure was moved from the main manuscript (previously Figure 2) following editorial reorganisation; the underlying numerical estimates are reported in Table 1 of the main manuscript and in Supplementary Table S3.

*[The composite multi-panel image of all 13 regional joinpoint plots is provided separately as Supplementary Figure S1 high-resolution image file at 300 dpi.]*

# **Index of supplementary tables (provided in separate .xlsx workbook)**

**Supplementary Table S1.** Annual age-standardized tuberculosis incidence rates (ASR per 100,000) for Thailand’s 13 health regions (2012–2023), standardized to the WHO World Standard Population (2000–2025).

**Supplementary Table S2.** Sensitivity analysis of zero-case +0.5 correction: comparison of regional AAPC estimates under four zero-handling methods (no correction, +0.5 [primary], +1.0, Bayesian gamma–Poisson).

**Supplementary Table S3.** Joinpoint sensitivity analysis: BIC-optimal joinpoint number and locations for all 13 health regions across nine combinations of maximum-joinpoints (1, 2, 3) and minimum-observations-per-segment (2, 3, 4).

**Supplementary Table S4.** Quantitative concordance between joinpoint and GAM-derived AAPC by health region (R², MAE, AAPC difference).

**Supplementary Table S5.** Effect-size–and–significance classification of all 77 provinces and ASR-based COVID-19 impact sensitivity analysis at ±5%, ±10% and ±15% thresholds.

**Supplementary Table S6.** Annual Global Moran’s I for provincial ASR (2012–2023) under queen, rook, k-nearest-neighbour (k = 4, 6, 8) and 200-km distance-band weights, with Benjamini–Hochberg FDR-adjusted P-values.

**Supplementary Table S7.** List of the 13 LISA-significant provinces with FDR-adjusted local Moran P-values and cluster classification.
